# Women living with their mothers-in-law

**DOI:** 10.12688/gatesopenres.13433.1

**Published:** 2021-11-17

**Authors:** Kristin E Bietsch, Katherine H LaNasa, Emily Sonneveldt

**Affiliations:** 1 Avenir Health, Glastonbury, CT, 06033, USA; 2 Tulane University, New Orleans, LA, 70118, USA

**Keywords:** Mother-in-law, Daughter-in-law, Decomposition analysis, Global trends

## Abstract

Background

Many studies have documented the impacts mothers-in-law have on daughters-in-law living in the same household, but few have quantified the scale of this co-residence. ****This study aims to estimate the proportion of married women living with their mothers-in-law across countries and time.

Methods

Using household rosters from 250 Demographic and Health Surveys in 75 countries, this paper uses the “relationship to head of household” question to identify households where married women live with their mothers-in-law.  For select countries with large changes, we decompose changes in rates into changes in the age structure of married women and the rate of women living with their mothers-in-law by age.

Results

This paper finds large variation in family structure around the globe, from 1% of married women in Rwanda to 49% in Tajikistan living with their mother-in-law.  Many countries with high co-residence in the 1990s continue to see high and increasing numbers today, especially in Central and Southern Asia, while some North and sub-Saharan African countries experienced substantial declines.  Decomposing changes by age and rates shows that changes in the age structure of married women is not driving changes in co-residence, but rather the rates are changing across age groups.

Conclusions

These findings show the large variation in women living with their mothers-in-law across the globe.  The authors provide publicly available code and future research ideas to encourage others to further our understanding of the impact of living with her mother-in-law on a woman’s life.

## Background

In patriarchal societies there are often expectations for a woman to live with her husband’s family after marriage. Kandiyoti (1988) identified the patrilocal extended household as a key element to the operation of ‘classic patriarchal systems’, which are most often found stretching from the Mediterranean to the Pacific, also termed the ‘mother-in-law belt’ ( Ember & Ember, 2003). This practice may be further perpetuated by modern labor migration where a woman’s husband leaves the home for extended periods of time and sends remittances to his family, thereby necessitating the woman to live with her in-laws for support during his absence ( Galam, 2017; Jama-Shai *et al.*, 2017).

 Women typically enter their new household with little status or power and their mother in-law, as the senior woman in the house, gains higher social status and more decision-making authority ( Kandiyoti, 1988). In Nepal, women reported they always were the last to eat in the household and sometimes went without food at the direction of their mother-in-law as punishment, even during pregnancy ( Pun *et al.*, 2016). Women have also reported their mother-in-law is a primary decision maker in their healthcare decisions ( Acharya *et al.*, 2010; Ganle *et al.*, 2015), including needing their mother-in-law’s permission to leave the house and visit a health center ( Hyder *et al.*, 2007).

A number of studies have identified the mother-in-law as a gatekeeper or barrier for accessing maternal health services for women ( Gupta *et al.*, 2015; Pun *et al.*, 2016). A study in Mali found that maternal health behaviors of women were strongly associated with the preferences of their mother-in-law ( White *et al.*, 2013). For example, women whose mother-in-law reported negative attitudes towards delivering in a health facility were less likely to receive care in a facility regardless of the woman’s own beliefs towards traditional birthing practices ( White *et al.*, 2013). Talbert *et al.* (2016) explained that women in Kenya felt pressure to adhere to the breastfeeding advice from their mother-in-law because they lived together and were expected to always obey.

A handful of studies in India have reported some positive effects for women living with their mother-in-law. Varghese & Roy (2019) found that women who lived with their mother-in-law during pregnancy were significantly less likely to experience severe maternal anemia and more likely to take regular iron supplements. Another study evaluated the impact of an intervention to improve the mother-daughter-in-law relationship and found a positive relationship with the mother-in-law protecting against violence from a woman’s husband ( Krishnan *et al.*, 2012).

To date, most published literature stems from qualitative research ( Hyder *et al.*, 2007; Jama-Shai *et al.*, 2017; Krishnan *et al.*, 2012; Nasrullah *et al.*, 2015) with limited quantitative data. Studies which do include quantitative data have used a variety of methods for measuring co-residence status with the mother-in-law, from primary surveys which ask coresident status directly ( Falb *et al.*, 2013; Falnes *et al.*, 2011; Gupta *et al.*, 2012; Jewkes *et al.*, 2019; White *et al.*, 2013), to estimates from national surveys ( Kiros & Kertzer, 2000) and census data ( Gibson & Mace, 2005; Huber *et al.*, 2017). Previous research has taken advantage of household schedules to identify mother-in-law/daughter-in-law cohabitation ( Speizer *et al.*, 2015; Varghese & Roy, 2019). It is also unknown how the globally increasing age of marriage ( Liang *et al.*, 2019) has impacted trends in co-residency.

In order to gauge the extent to which married women around the world live with their mothers-in-lay, there remains a need for an established quantitative method for measuring coresident status across countries and time using publicly available data. Research focusing on the health and well-being of daughters-in-law could be greatly expanded if researchers were able to use the large datasets collected by nationally representative surveys. Research could span demographic, economic, health, and many other fields, documenting both how living with a mother-in-law impacts daughters-in-law, but also how this relationship has changed over time and varies across settings.

## Methods

This paper measures co-resident status with mothers-in-law using widely available data to compare trends across time and region. The Demographic and Health Surveys (DHS) program collects nationally representative data on a range of health topics approximately every five years in most countries, making it a useful survey to examine the health status within and across countries and time. Using data available from the household schedules of 250 surveys from 75 countries we are able to estimate the share of married women who live with their mothers-in-law, compare prevalence between countries and regions, and examine changes over time. For select countries with large changes we decompose changes in co-residence into changes in co-residence by age and changes in the age structure of married women. 

To identify relationships between household members in the DHS, we used household schedules. As part of the household questionnaire, an interviewer asks one member of the household to list all usual members and visitors and collects data on each person’s age, sex, relationship to household head, and other information ( DHS). While a household schedule was collected as part of Phase 1 of the DHS (1984–1989), relationship to head of household was introduced in Phase 2 (1988–1993) ( Institute for Resource Development/Macro International, Inc, 1990). 258 surveys have available data for Person Recode files which are constructed from the household questionnaire with each member of a household as his or her own row. 

Using the Person Recode files, we identified the labels and values for possible responses to the question of relationship to the household head. These relationships were then cleaned and classified into one of the following categories: head, wife or husband, son or daughter, son-in-law or daughter-in-law, parent, parent-in-law, sibling, grandparent, grandchild, adopted child, other relative, not related, and other. During data cleaning, one survey was removed from the analysis because it did not contain labels for DHS variable “hv101,” the variable containing the relationship to head of household, (Jordan 1990) and seven surveys were excluded because they did not differentiate between parents and parents-in-law (Bolivia 2003, Dominican Republic 1996, Dominican Republic 2002, Dominican Republic 2007, Dominican Republic 2013, Nicaragua 2001, and Peru 1992). Because relationships are only recorded in reference to the household head, we created a relationship matrix to identify the potential relationships between two people based on their relationship to the head ( Table 1). 

**Table 1.** Relationship matrix.

Index	Head	Wife or husband	Son or daughter	Son-in-law or daughter-in- law	Parent	Parent-in- law	Sibling	Grandchild	Adopted child	Other relative	Not related	Other
**Head**	Self	Spouse	Parent	Parent-in-law	Child	Son-in- law or daughter- in-law	Sibling	Grandparent	Other	Other	Other	Other
**Wife or** **husband**	Spouse	Self	Parent	Parent-in-law	Son-in- law or daughter- in-law	Child	Sister-in-law or brother- in-law	Grandparent	Other	Other	Other	Other
**Son or ** **daughter**	Child	Child	Sibling	Spouse or sister-in-law or brother-in-law	Grandchild	Grandchild	Niece or nephew	Parent or aunt/uncle	Other	Other	Other	Other
**Son-in-law or** ** daughter-in-** **law**	Son-in-law or daughter- in-law	Son-in-law or daughter- in-law	Spouse or Sister-in-law or brother-in-law	Sister-in-law or brother-in-law	Grandchild- in-law	Grandchild- in-law	Nephew- or niece-in-law	Parent or aunt/uncle	Other	Other	Other	Other
**Parent**	Parent	Parent-in- law	Grandparent	Grandparent-in- law	Spouse	in-law (parents of married children)	Parent	Great grandchild	Other	Other	Other	Other
**Parent-in-law**	Parent-in- law	Parent	Grandparent	Grandparent- in-law	in-law (parents of married children)	Spouse or both parents-in- law	Other in-law	Great Grandparent	Other	Other	Other	Other
**Sibling**	Sibling	Sister-in-law or brother- in-law	Aunt or uncle	Aunt- or uncle- in-law	Child	Other in- law	Sibling	Great aunt or uncle	Other	Other	Other	Other
**Grandchild**	Grandchild	Grandchild	Parent or aunt/uncle	Parent or aunt/uncle	Great grandchild	Great grandchild	Great Niece or nephew	Sibling or cousin	Other	Other	Other	Other
**Adopted child**	Other	Other	Other	Other	Other	Other	Other	Other	Other	Other	Other	Other
**Other relative**	Other	Other	Other	Other	Other	Other	Other	Other	Other	Other	Other	Other
**Not related**	Other	Other	Other	Other	Other	Other	Other	Other	Other	Other	Other	Other
**Other**	Other	Other	Other	Other	Other	Other	Other	Other	Other	Other	Other	Other

For each household member, we created a variable with the household identification information, line number, age, and sex of the household member. Transforming this data from long to wide created a dataset where each line represents a household, and each column is a single member. This dataset was then merged to the original Person Recode file, so that each member of a household also contained information on all the other household members. This file was reshaped from wide to long, resulting in dyads of two household members as observations. Finally, the relationship matrix was used to identify the relationship of the two members in the dyad.

To identify mother-in-law/daughter-in-law cohabitation we selected all female residents between the ages of 15 and 49, in order to match the age range of women eligible for the individual questionnaire. We classified a woman as living with their mother-in-law if they had a dyad relationship where they are the “son-in-law or daughter-in-law” of the other member and if the sex of the other member of the dyad is female. We merged this information with the Individual Recode file (women 15-49) and selected currently married women. It was necessary to include the Individual Recode file because not all household schedules recode marital status. 

With the Individual Recode file we calculated the percent of married women who live with their mothers-in-law in total and by age group. For four example countries, we constructed a decomposition of two rates ( Kitagawa, 1955) to determine if changes over time are attributable to changes in the age structure of married women or changes in the rate schedule by age.

All code used to transform household rosters and individual recode files into mother-in-law analysis and figure is written in R using Rstudio version 1.2.5019 and is archived on Zenodo ( Bietsch, 2021). The code uses multiple packages which need to be installed in R; the full list of packages and links to their Cran documentation is available on the author’s GitHub.

### Ethics and consent

 Demographic Health Surveys are available on The DHS Program’s website. Due to the use of secondary data, ethical approval was not needed for this study. Procedures and questionnaires for DHS surveys are approved by the ICF Institutional Review Board. More information about the DHS’s informed consent and privacy regulations can be found on their website.

## Results

### Current Levels and Trends

**Figure 1.**
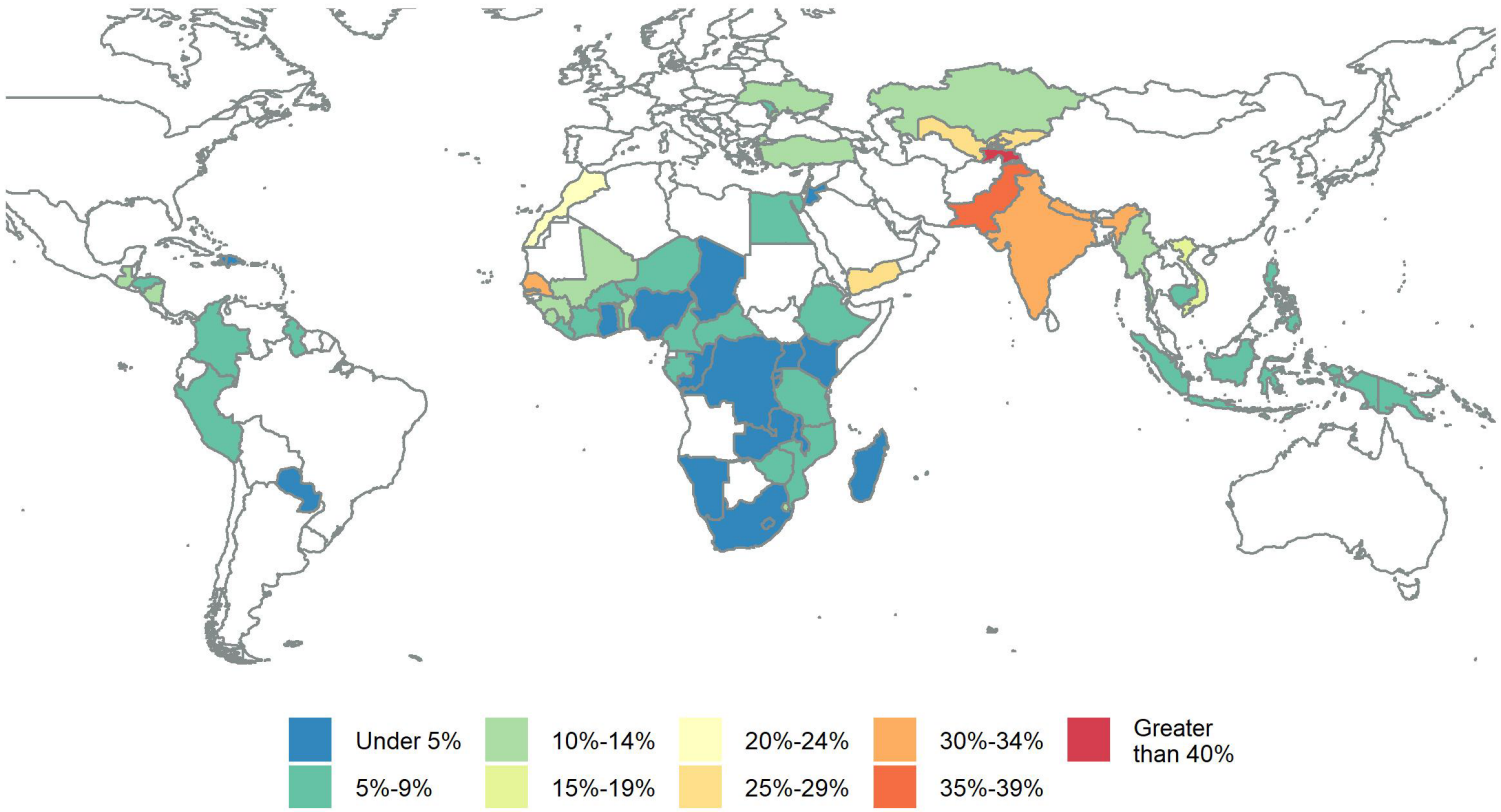
Map of the proportion of married women living with their mother-in-law countries with The Demographic and Health Surveys data.

For the most recent survey in 75 countries with available data, Figure 1 maps the proportion of women living with their mother-in-law. In 23 countries (Angola, Bolivia, Brazil, Burundi, Chad, Comoros, Congo, Democratic Republic of Congo, Dominican Republic, Ghana, Haiti, Jordan, Kenya, Madagascar, Malawi, Namibia, Nigeria, Paraguay, Rwanda, Sao Tome and Principe, South Africa, Uganda, and Zambia), less than 5% of women live with their mother-in-law. Co-residence of over 30% is found in nine countries (Afghanistan, Albania, Armenia, Azerbaijan, India, Nepal, Pakistan, Senegal, and Tajikistan), and in Armenia and Tajikistan more than 40% of married women were living with their mother-in-law.

### Regional trends

In Central and Southern Asia, five countries (Afghanistan, India, Nepal, Pakistan, and Tajikistan) reported more than 30% of women living with their mother-in-law. Of the eight countries with data available from multiple years (Bangladesh, India, Kazakhstan, Kyrgyz Republic, Maldives, Nepal, Pakistan, and Tajikistan), the mean percent of women living with their mother-in-law has statistically increased over time in six (Bangladesh, India, Kyrgyz Republic, Nepal, Pakistan, and Tajikistan). The largest increase occurred in Pakistan, from 29.6% in 1990-91 to 35.8% by 2017-18. In Tajikistan, nearly half (48.5%) of married women reported they lived with their mother-in-law in 2017; this was the highest reported proportion in this region and among all countries included in this analysis.

In Eastern and South-Eastern Asia, three out of the six countries (Myanmar, Timor-Leste, and Vietnam) included in this study reported more than 10% of women lived with their mother-in-law. Of countries with trend data, Cambodia, Indonesia, and the Philippines all saw significant increases between their earliest and most recent surveys.

In the region of North Africa, Western Asia and Europe, five out of ten countries (Albania, Armenia, Azerbaijan, Morocco, and Yemen) reported more than 20% of married women lived with their mother-in-law. Among the seven countries in this region with data available from multiple survey years (Albania, Armenia, Egypt, Jordan, Morocco, Turkey, and Yemen), the proportion of women living with their mother-in-law increased in only one country, Armenia. Egypt, Jordan, Morocco, and Turkey all showed significant decreases. 

In sub-Saharan Africa, only Gambia and Senegal reported more than 20% of women lived with their mother-in-law. Among the 33 countries with data available from multiple years (Benin, Burkina Faso, Burundi, Cameroon, Chad, Comoros, Congo, Congo Democratic Republic, Cote d'Ivoire, Ethiopia, Gabon, Gambia, Ghana, Guinea, Kenya, Lesotho, Liberia, Madagascar, Malawi, Mali, Mozambique, Namibia, Niger, Nigeria, Rwanda, Senegal, Sierra Leone, South Africa, Tanzania, Togo, Uganda, Zambia, and Zimbabwe), the proportion of women living with their mother-in-law statistically decreased in 21 countries (Benin, Burkina Faso, Cameroon, Chad, Congo, Congo Democratic Republic, Cote d'Ivoire, Gabon, Kenya, Lesotho, Madagascar, Malawi, Namibia, Niger, Nigeria, Sierra Leone, South Africa, Tanzania, Togo, Uganda, and Zambia), showed no change in seven countries (Burundi, Comoros, Ethiopia, Gambia, Guinea, Liberia, and Zimbabwe), and statistically increased in five countries (Ghana, Mali, Mozambique, Rwanda, and Senegal).

In Latin America and the Caribbean, less than 15% of married women reported living with their mother-in-law in all 11 countries (Bolivia, Brazil, Colombia, Dominican Republic, Guatemala, Guyana, Haiti, Honduras, Nicaragua, Paraguay, and Peru). Among the eight countries with trend data, three countries statistically increased (Colombia, Guatemala, and Haita), although the changes were all less than three percentage points. Two countries saw statistically significant declines (Brazil and Peru), and three had no change (Bolivia, Dominican Republic, and Honduras). There was only one country from Oceania included in this analysis, Papa New Guinea. In this country, 8.4% of women reported living with their mother-in-law in 2016.

### Age groups

For the most recent surveys in all countries with data, 12.1% of married women in the countries selected for this study live with their mother-in-law. When stratified by age groups, most women living with their mother-in-law are 15–19 years (28.4%), followed by women ages 20–24 (21.6%). Table 2 presents the age-stratified proportion of married women living with their mother-in-law in each region for the most recent available surveys. In Central and Southern Asia, over half of women 15–24 live with their mother-in-law. In North Africa/Western Asia/Europe countries, nearly half of all married women ages 15–19 lived with their mother-in-law and over a third of women ages 20–24 lived with their mother-in-law. In Eastern and South-Eastern Asia, over one thirds (33.8%) of married women ages 15–19 and over a quarter (26.4%) of women ages 20–24 live with their mother-in-law. In sub-Saharan Africa, Latin America and the Caribbean, and Oceania, less than 20% of married women ages 15–19 live with their mother-in-law.

**Table 2.** Proportion of married women living with their mother-in-law by age group.

	Age Group						
	**15–19**	**20–24**	**25–29**	**30–34**	**35–39**	**40–44**	**45–49**
Total	28.4%	21.6%	15.5%	10.9%	7.7%	5.4%	3.8%
**Region**							
Central and Southern Asia	58.6%	52.1%	39.3%	27.0%	16.7%	10.6%	6.7%
Eastern and South-Eastern Asia	33.8%	26.4%	16.7%	10.2%	7.2%	4.9%	3.9%
Latin America & Caribbean	19.5%	11.7%	6.6%	4.2%	2.8%	2.4%	1.7%
North Africa/West Asia/Europe	46.2%	36.1%	28.6%	20.6%	15.8%	11.1%	7.6%
Oceania	17.7%	15.6%	10.5%	7.5%	5.7%	3.1%	2.2%
Sub-Saharan Africa	17.5%	11.8%	8.2%	6.1%	4.6%	3.4%	2.6%

### Decomposition analysis

Egypt, Nepal, Senegal, and Turkey were selected for the decomposition analysis because of their large increases (Nepal and Senegal) and large declines (Egypt and Turkey) ( Table 3). In Egypt, the proportion of women living with their mother-in-law decreased by 12.1 percentage points between 1992 and 2014. The majority of this change was driven by a 12.0 percentage point decrease in the rate of women living with their mother-in-law, over half of which was among women ages 20–29 (-7.62 combined percentage points). The total contribution of changes in married age structure was minimal (0.1 percentage point).

**Table 3.** Decomposition of the rates of women married and living with their mother-in-law.

Country	Change in proportion of women living with mother-in-law	Contribution from the rate of women married	Contribution from the rate of women living with mother-in-law
**Egypt**	**-12.12**	**-0.12**	**-12.00**
*Age Group Contribution*			
*15–19*		*-0.306*	*-1.531*
*20–24*		*0.026*	*-3.802*
*25–29*		*0.218*	*-3.816*
*30–34*		*0.053*	*-1.944*
*35–39*		*-0.123*	*-0.898*
*40-44*		*-0.045*	*-0.090*
*45–49*		*0.055*	*0.082*
**Nepal**	**3.21**	**-0.99**	**4.20**
*Age Group Contribution*			
*15–19*		*-0.610*	*0.447*
*20–24*		*-0.640*	*1.108*
*25–29*		*-0.035*	*1.272*
*30–34*		*0.052*	*0.832*
*35–39*		*0.018*	*0.141*
*40–44*		*0.087*	*0.223*
*45–49*		*0.138*	*0.174*
**Senegal**	**10.61**	**-0.41**	**11.02**
*Age Group Contribution*			
*15–19*		*-0.871*	*0.427*
*20-24*		*-0.156*	*2.461*
*25–29*		*0.169*	*3.267*
*30–34*		*0.422*	*2.696*
*35–39*		*0.084*	*1.068*
*40–44*		*-0.205*	*0.756*
*45–49*		*0.150*	*0.345*
**Turkey**	**-9.00**	**-3.82**	**-5.18**
*Age Group Contribution*			
*15–19*		*-1.999*	*-0.309*
*20–24*		*-2.585*	*-1.524*
*25–29*		*-0.317*	*-1.763*
*30–34*		*0.133*	*-0.681*
*35–39*		*0.429*	*-0.574*
*40–44*		*0.297*	*-0.435*
*45–49*		*0.225*	*0.106*

In Nepal, the proportion of women living with their mother-in-law increased by 3.21 percentage points between 2011 and 2016. This change was driven entirely by the increasing rate of women living with their mother-in-law (4.2 total percentage points), particularly among women ages 20–24 (1.11 percentage points) and 25–29 (1.27 percentage points). Changes in the age structure of married women contributed negative one percentage points, which was driven by fewer women ages 15–19 (0.61 percentage points) and 20-24 (0.64 percentage points) married in 2016.

In Senegal, the proportion of women living with their mother-in-law increased by 10.61 percentage points between 1993 and 2019. An increase in the rate of women living with their mother-in-law (11.0 total percentage points) created to this change, mainly due to increases among women ages 20–34 (8.42 combined percentage points). Although changes in the age structure would have led to a decline in women living with their mother-in-law if the rates had not changed, this contribution was small (-0.4 percentage points).

In Turkey, the proportion of women living with their mother-in-law decreased by 9.00 percentage points between 1993 and 2013. The contributions of the rate of women living with their mother-in-law (-5.2 percentage points) and the rate of women married (-3.8 percentage points) were similar. The largest contributor for changes in rates was among women ages 20–29 (-3.29 combined percentage points). The largest decline in age structure was from married women aged 15–24 (4.59 combined percentage points).

## Discussion

While no overarching global pattern emerges, many interesting regional trends appear from this analysis. In Central and Southern Asia, which had some of the highest rates of co-residence in the 1990s, most countries have seen significant increases in co-residence. Nepal’s recent large increase in co-residence coincides with large scale migration of husbands to foreign countries for work. However, when looking at the remainder of Ember and Ember’s ‘mother-in-law belt,’ we see a decline in women living with their mothers-in-law. This is particularly true in Egypt and Turkey, which have had large declines, not caused by the age structure of the married population but by declines in daughters-in-law living with their mothers-in-law. Most countries in sub-Saharan Africa are also experiencing a large decline in co-residence, though co-residence was never as common as in other regions. 

Given the global variation in the proportion of women living with their mother-in-law and the limited research which has accounted for this factor, there remain many future directions for research. Future studies may assess the timing of childbearing. Furthermore, does living with their mother-in-law influence the type of contraception a woman uses? As prior studies have reported some women need permission from their mother-in-law to leave the house ( Hyder *et al.*, 2007; Rew *et al.*, 2013), this could impact her ability to visit a family planning clinic or resupply short-term methods. Other studies have reported a woman’s mother-in-law has decision-making power over a daughter-in-law’s health ( Acharya *et al.*, 2010; Ganle *et al.*, 2015), therefore future studies may determine whether living with a mother-in-law influences who is the primary family planning decision-maker. Finally, how does living with the mother-in-law impact a woman’s perceived autonomy and empowerment within the household, the relationship with her husband, and in her healthcare decisions? 

 One limitation in this research is that we are unable to identify mother-in-law residence in some households that contain several branches of a family co-residing. Because we are identifying in-laws through the household head, if the household head is the not directly related to the mother-in-law or daughter-in-law, in some cases the relationship cannot be established. For example, looking at the relationship matrix in Table 1, if either member is identified as “other relative” then a mother-in-law relationship cannot be established. We believe that these cases will be infrequent, but may cause an undercount of co-residence.

## Conclusion

By creating a methodology for studying household dyad relationships, we have established a means of identifying mother-in-law co-residence for women in Demographic and Health Surveys. We have found global change in the number of women living with their mothers-in-law; in some regions this trend is increasing, and in others it is decreasing. In countries with the largest change, the change is overwhelmingly caused by changes in the rates of women cohabiting, not in the age structure of married women. Our goal in this research was to shed light on how many women live with their mothers-in-law and produce replicable code to allow other research to continue to explore how co-residence impacts women’s lives in a myriad of ways.

## Data availability

### Underlying data

This study uses secondary data from the Demographic and Health Surveys which provide anonymized data to researchers. The Demographic and Health Surveys are available from https://dhsprogram.com/ at no cost for academic research. 

Users must register to request and download dataset at https://dhsprogram.com/data/new-user-registration.cfm. After approval, datasets can be downloaded from https://dhsprogram.com/data/Using-Datasets-for-Analysis.cfm.

Datasets are available as SAS, Stata, SPSS, and Flat Ascii files. 

Zenodo: kristinbietsch/MIL-Analysis: Release for Gates 110921 v2. https://doi.org/10.5281/zenodo.5659783. ( Bietsch, 2021)

This project contains the following files:

- Age Distribution of Married Women.R
- Age Distribution of Married Women.csv
- Country Regions.csv
- Data Key
- Data_MIL061821.csv
- Data_MILAGE061821.csv
- HV101Coding_Cleaned.csv
- HV116 Coding.R
- Household Member Coding Loop 061421.R
- ISOListFull.csv
- LICENSE
- License CC-BY-4
- Loop MIL Code 072621.R
- MIL Code for Surveys that do not run through loop.R
- MIL Decomposition 081921.R
- Master DHS Survey List.xlsx
- Mother In Law Further Analysis.R
- Packages Used in MIL analysis.xls
- Relationship Matrix.csv
- ne_50m_admin_0_countries.shp

Data are available under the terms of the Creative Commons Attribution 4.0 International license (CC-BY 4.0). 

Code is available under an MIT license.

The code to reproduce the analysis is also available on GitHub: https://github.com/kristinbietsch/MIL-Analysis
