# Supplementary material for: Women living with their mothers-in-law
Source: Gates Open Res. 2021 Nov 17;5:170. [Version 1] doi: 10.12688/gatesopenres.13433.1 (PMC8649626; doi:10.12688/gatesopenres.13433.1)
Supplement: Peer review history

# Peer review: Reviewer response for version 1

**DOI:** 10.21956/gatesopenres.14688.r31419

**Published:** 2021-12-09

**Authors:** Aysun Hiziroglu Aygun

**Affiliations:** 1 Department of Economics, İstanbul Teknik Üniversitesi, İstanbul, Turkey

In this article, Bietsch, LaNasa and Sonneveldt provide quantitative evidence from 75 countries on the co-residence of married women with mother-in-laws. A clearly written review of the literature is also provided to support the motivation for such an investigation. The authors' main finding is a broad statement of the shares of women who live with their mother-in-law across the globe. Also regional statistics are available for these shares by age group.

 Here are some comments that I believe would contribute to the presentation of the findings.

- It will be interesting to see the birth cohorts table/statistics along with age groups. This would be more informative in terms of understanding the time trend in co-residence.
- For the limitations of the study, the authors state that they could not identify the relationship in some of the households. Observing that a lot of African countries have low rates of co-residence, one wonders if this has to do with the data limitation. In other words, could the unidentified relationship be more common in Africa?
- It will be nice to add notes to the tables and figures referring to the data sources: i.e. is it the aggregated DHS or the most recent year?
- I think the decomposition analysis is interesting and could be used for more than four countries (maybe for each region).

Is the work clearly and accurately presented and does it cite the current literature?

Yes

If applicable, is the statistical analysis and its interpretation appropriate?

Yes

Are all the source data underlying the results available to ensure full reproducibility?

Yes

Is the study design appropriate and is the work technically sound?

Yes

Are the conclusions drawn adequately supported by the results?

Yes

Are sufficient details of methods and analysis provided to allow replication by others?

Yes

Reviewer Expertise:

Applied Microeconomics, Labor Economics, Health Economics

I confirm that I have read this submission and believe that I have an appropriate level of expertise to confirm that it is of an acceptable scientific standard.

# Peer review: Reviewer response for version 1

**DOI:** 10.21956/gatesopenres.14688.r31423

**Published:** 2021-12-06

**Authors:** Momoe Makino

**Affiliations:** 1 Institute of Developing Economies, The Japan External Trade Organization (JETRO), Chiba, Japan

The mother-in-law and daughter-in-law relationship is understudied in household economics, which has almost exclusively focused on the husband-wife relationship in intrahousehold bargaining. However, given that the mother-in-law can influence the daughter-in-law’s behavior and decision-making, even more than the latter’s husband, there should be a rich scope to be explored in this mother-in-law and daughter-in-law relationship. This paper can be a springboard for household economists to investigate this relationship.

 Household economists may not consider the descriptive statistics that the authors presented in this study as the results, but the descriptive statistics are still informative given the understudied relationship of mother-in-law and daughter-in-law. I found it interesting that the mother-in-law and daughter-in-law cohabitation has increased in Central and South Asian countries and has decreased in Sub-Saharan African countries. Besides, the fact that the mother-in-law and daughter-in-law cohabitation has increased in its most prevalent region seems interesting because nuclear families have increased in most developed countries. Exploring the reasons explaining the difference in cohabitation trend across regions may be one potential topic.

 The authors’ suggestion concerning potential future topics based on the descriptive statistics also stimulate researchers. In particular, the impact of mother-in-law and daughter-in-law cohabitation on women’s empowerment and intrahousehold resource allocation will attract household economists’ attention.  

 As for minor comments, Table 1 does not seem to be very informative as the relationship matrix simply shows the relationship of two members in the dyad. By contrast, the description about how the dyad was made (the second paragraph in p.4) is informative. The current Table 1 seems also confusing given this description in the second paragraph because the description may remind readers of the table of the dataset, i.e., each row is a member of the household, who is matched to another member of the household.

Is the work clearly and accurately presented and does it cite the current literature?

Yes

If applicable, is the statistical analysis and its interpretation appropriate?

Yes

Are all the source data underlying the results available to ensure full reproducibility?

Yes

Is the study design appropriate and is the work technically sound?

Yes

Are the conclusions drawn adequately supported by the results?

Yes

Are sufficient details of methods and analysis provided to allow replication by others?

Yes

Reviewer Expertise:

Household Economics, Development Economics

I confirm that I have read this submission and believe that I have an appropriate level of expertise to confirm that it is of an acceptable scientific standard.

# Peer review: Reviewer response for version 1

**DOI:** 10.21956/gatesopenres.14688.r31424

**Published:** 2021-11-24

**Authors:** Harihar Sahoo

**Affiliations:** 1 Department of Family & Generations, International Institute for Population Sciences, Mumbai, Maharashtra, India

The estimation of the proportion of married women living with their mothers-in-law across countries and time is a new addition to the existing literature. The present study uses appropriate data to address the issue. The authors have considered appropriate methods. The study findings highlight the scope for further study in future.

Is the work clearly and accurately presented and does it cite the current literature?

Yes

If applicable, is the statistical analysis and its interpretation appropriate?

Yes

Are all the source data underlying the results available to ensure full reproducibility?

Yes

Is the study design appropriate and is the work technically sound?

Yes

Are the conclusions drawn adequately supported by the results?

Yes

Are sufficient details of methods and analysis provided to allow replication by others?

Yes

Reviewer Expertise:

Family Demography

I confirm that I have read this submission and believe that I have an appropriate level of expertise to confirm that it is of an acceptable scientific standard.
